# Fish consumption during menarche, menstruation, pregnancy and postpartum in Sikuani women from Meta, Colombia

**DOI:** 10.1186/s13002-019-0326-z

**Published:** 2019-10-10

**Authors:** Luisa Fernanda Cubillos-Cuadrado, Daniela Stephany Muñoz-Hernández, Carlos Alberto Vásquez-Londoño

**Affiliations:** Faculty of Medicine, Universidad Nacional de Colombia, Bogotá, Colombia

**Keywords:** Food avoidance, Sikuani, Menarche, Menstruation, Pregnancy, Postpartum, Fish Consumption

## Abstract

**Background:**

Societies have selected their food for health, cultural, religious, political, economical, and environmental reasons. Most of the food included in Sikuani traditional diet still comes from wild natural resources and involves numerous species of fish, mammals, birds, reptiles, amphibians, insects, and plants. During certain periods of the Sikuani women’s reproductive cycle, fish intake is avoided. The objective of this research is to study the conceptions underlying fish consumption regulations among Sikuani women at the Wacoyo Reservation, in Meta, Colombia.

**Methods:**

We conducted a field study through interviews and participant observation with Sikuani Indigenous from the Wacoyo Reservation (Colombia). We inquired about the conceptions of fish consumption regulation by Sikuani women during the stages of the reproductive cycle. PCA (principal component analysis) was used to identify the most important characteristics of fish that are related to the avoidance of fish intake by Sikuani women during pregnancy. This study combines qualitative and quantitative analysis.

**Results:**

It was found that during menarche and postpartum fish consumption is avoided by Sikuani women only before the ritual known as the prayer of the fish is performed. The menstruation does not imply significant regulations for fish intake, while during pregnancy there are multiple and specific avoidances for the consumption of fish. According to our results, there are some features of fish associated with their regulation on the diet of pregnant Sikuani women. The consumption of some fish is avoided during pregnancy because it is related to the appearance of disease caused by *ainawi*, protector spirits of aquatic animals.

**Conclusions:**

The traditional diet of Sikuani women includes numerous fish species and an important proportion of them are avoided during menarche, menstruation, gestation, and postpartum. According to our results, there are some features of fish associated with their regulation on the diet of pregnant Sikuani women. The main reasons underlying the avoidance of fish consumption by Sikuani women are the prevention of human disease as well as the strengthening of communities and ecosystems resilience.

## Background

Eating is a biological need and a human experience that is articulated in social, economic, ecological, medical, and cultural dimensions. Food systems are reflections of culture, representations of worldviews and constitute a heritage, since they are the result of historical processes of memory and identity of people [1, 2]. Societies select their food according to the availability of natural resources, its accessibility in territories with dynamic environmental and social conditions, and also in consonance to diverse and complex conceptual frameworks regarding nutrition, food classification, preferences, and restrictions [3, 4]. Food avoidance can be due to cultural, ecological, health, magical-religious or economic reasons, choices observed in everyday life and in specific events such as festivals, religious celebrations, initiation rituals, hunting expeditions, and funerals, as well as during key moments of the human life cycle such as menarche, menstruation, pregnancy, lactation, and postpartum [5]. Some anthropological theories propose that food taboos could work to preserve the environment by restricting resource consumption, strengthening group cohesion, and cultural identity, as well as identifying and protecting from dangerous foods [6]. Some anthropologists state that foods derived from animals are more commonly restricted than plant-based foods [7]. In the case of the Sikuani ethnic group from South America, the regulations for food intake during a woman’s life cycle play an important role in their social organisation, territory management, and cultural expressions [8].

The Sikuani belong to the Guahibo linguistic family, they were originally nomads clustered in clans and inhabited historically the Colombian-Venezuelan plains [9]. Historically, the Sikuani have been displaced from their territories on several periods, during the 16–18th centuries by Spanish settlers who occupied the most fertile areas and confined indigenous people into small rural lands called *resguardos* (indigenous reservations), and later to the present by missionaries and armed groups [10, 11]. In Colombia, the Sikuani are currently located in the Orinoco basin and constitute the most numerous indigenous population of the eastern region of the country. Nowadays, the Sikuani reside in sedentary or semi-sedentary conditions along the Manacacías, Meta, Vichada, Guaviare, and Orinoco rivers, in indigenous reservations situated in savannah and jungle areas where they subsist from hunting, fishing, gathering, agriculture, and recently from other economic activities [12, 13]. Despite the notorious influences of forced displacement on the culture and territory management of Sikuani people, most of their dietary intake still comes from wild natural resources including numerous species of fish, mammals, birds, reptiles, amphibians, insects, and plants [14, 15].

According to the Sikuani worldview, nature belongs to spiritual entities called *ainawi* that inhabit water depths, forests, the underworld, and other landscapes [16], implying that the consumption of most animals by humans can cause disease unless an authorisation of the *ainawi* is requested by the *penajorrobinü* or Sikuani shaman through prayers, chants, and other rituals [17]. During crucial moments of Sikuani woman life cycle as the menarche, menstruation, pregnancy, and postpartum, the dietary intake of fish and other animals is avoided as its consumption is associated with different ailments caused by the *ainawi*, making necessary the performance of a ritual called the prayer of the fish [9, 18–21].

Despite the great diversity of species that still constitute the Sikuani traditional diet, the complexity of food restrictions associated to health and environmental protection, and its importance in their daily life and culture, the regulations for fish consumption during important moments of woman life cycle remain unexplored. The aim of this research is to study the reasons underlying the avoidance of fish consumption during menarche, menstruation, pregnancy, and postpartum of Sikuani women at the Wacoyo Reservation, in Meta, Colombia.

## Methods

### Study area

**Fig. 1:**
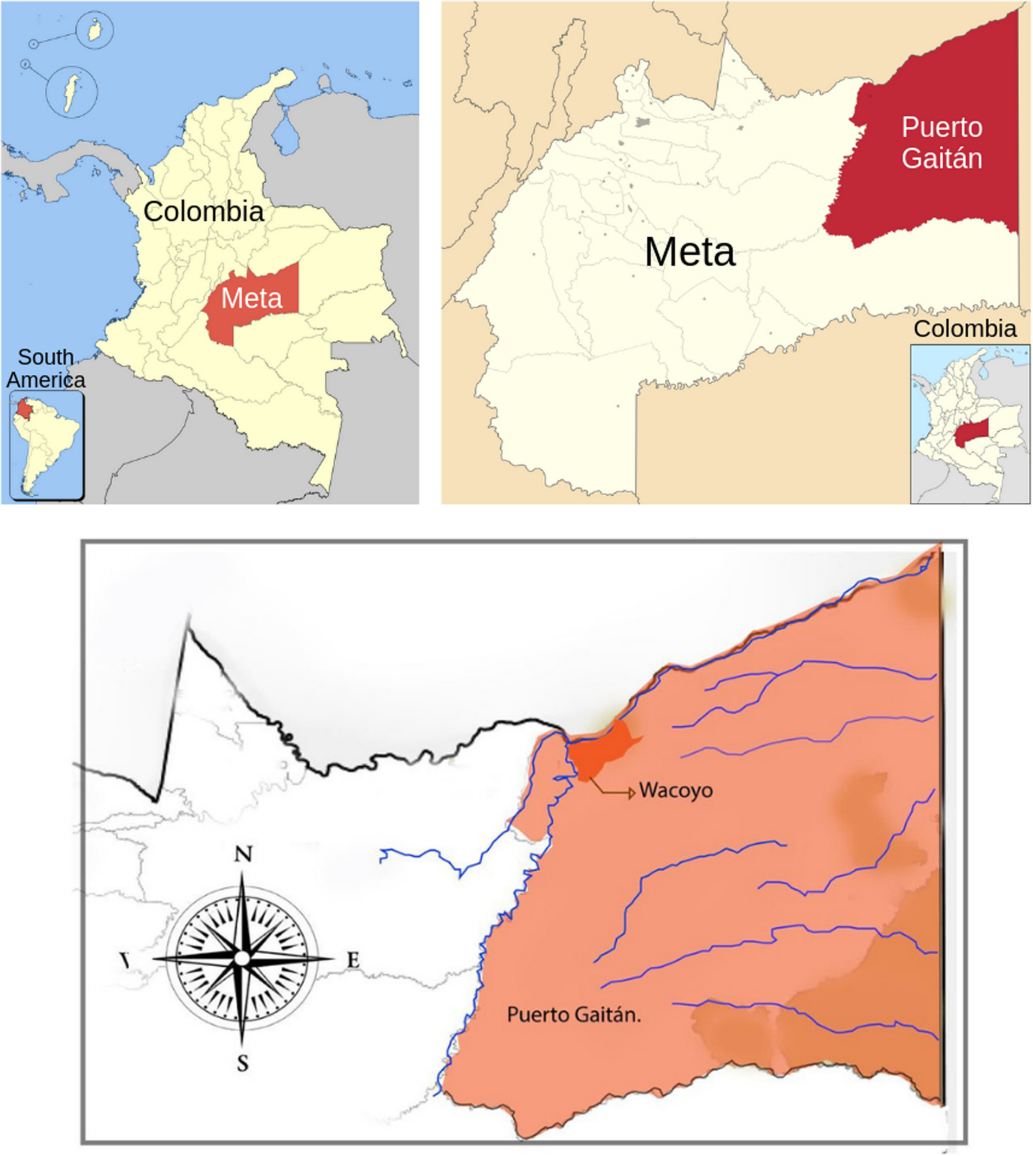
Location of Wacoyo indigenous reservation in Puerto Gaitán-Meta (orange). From left to right: South America, Colombia, Meta department, Puerto Gaitán, Wacoyo Reservation

The Wacoyo Reservation is located at the eastern region of Colombia on the Orinoco river basin, near to the municipality of Puerto Gaitán in the department of Meta, surrounded by the Manacacías and Meta rivers, at the coordinates 4°18′40′′ N and 72°04′46′′ W, with temperatures ranging from 20 °C to 35 °C (Fig. 1); it is considered a tropical forest [11]. Most of the inhabitants of Wacoyo Reservation belong to the Sikuani ethnic group and arrived in this land after being displaced from their ancestral territories. Despite the influence of centuries of sedentarisation and forced migration, the Sikuani people of Wacoyo have resisted and have been able to preserve their culture [11, 19].

According to the Sikuani inhabitants of Wacoyo, their predecessor clans were originated, in a mythical period, from totemic animals called *momowi* that were the first to populate the Orinoco basin. The current Wacoyo community was founded in 1939 by the Sikuani leader called Antonio Turriego Yepes, but only until the 1980s their land was legalised as a reservation and their indigenous organisations were recognised by the government [14]. In the present day, the Wacoyo Reservation has a local authorities headed by the “captain” who leads the organisation of the community and interacts with the local and national governments. According to the Indigenous Affairs Direction of the Colombian Ministry of Interior, the territory of Wacoyo Reservation has an area of 8200 hectares and in 2016 was inhabited by 1537 people [13]. One of the captains of Wacoyo reported that nowadays, the population of the reservation is approximately 2000 inhabitants. Due to its proximity to rivers, the reservation’s food supply is largely obtained not only from fishing, but also from hunting, wild fruits collection and cassava, plantain, and corn crops.

### Data collection

The information was collected from fieldwork during five visits to the Wacoyo Reservation between July 2016 and June 2017. First, the written permit of the community leaders was obtained. As the diet and food restrictions among Sikuani people are complex issues well known by traditional experts, they were considered as the informants for this study. Semi-structured interviews were conducted in 6 families, with a total of 20 people including midwives, traditional healers, leaders, and health promoters of Wacoyo Reservation. Interviews were done in order to inquire about food practices and conceptions during the life cycle stages of the Sikuani woman. Noting that the major food regulations and Sikuani rituals were related to fish consumption, we focused specifically into this topic. Interviewing was done until data saturation was noticed [22]. Additionally, the participant observation technique was used witnessing the daily life of six Sikuani families, observing the acquisition and preparation of traditional foods, as well as rituals like the prayer of the fish performed for a Sikuani girl after her menarche.

For the interviews, colour images of fish were used [23, 24]. One hundred fifty-eight fish species were selected from local fish inventories [25], informants were asked to identify the images of the species that are part of the diet of Sikuani women, if they were restricted during the menarche, menstruation, pregnancy, or postpartum and which fish features could have an association with the avoidance of its intake, including fish size (S), type of food (TF), sharp teeth (ST), fish that disguise (FD), skin plaques (PP), pointed mouth (PM), presence of barbels (PB), snake shape (SS), and big fins (BG).

### Data analysis

Recorded information during field trips was transcribed, then the data collected from each informant and the results of the participant observation were analysed qualitatively using data triangulation [26] and thematic analysis about the diet avoidance during different stages of the Sikuani woman life cycle and conceptions about fish. As data collected from interviews included multiple morphological characteristics of the fish to be correlated with avoidance of their consumption during pregnancy by Sikuani women, we used principal component analysis (PCA) to recognise the most important variables [27]. PCA was carried out using the *PAST* software [28].

## Results

### Fish in the diet of the Sikuani women

Participants recognised a total of 139 fish species consumed by Sikuani women. An important proportion of the fish species eaten by Sikuani women are avoided during menarche, menstruation, gestation, and postpartum, as shown in Table 1.

**Table 1:** Fish in the Sikuani diet during women’s vital cycle

Scientific taxa	Sikuani´s name	Is it eaten during pregnancy?	Is it eaten during menarche and postpartum?	Is it eaten during menstruation?
*Curimatopsis macrolepis*	*Bajuto/sisieto*	Yes	No	Yes
*Anchoviella guianensis*	*Wanaribajut*	Yes	No	Yes
*Prochilodus nigricans*		Yes	No	Yes
*Prochilodus mariae*	*Kutzato*	Yes	No	Yes
*Semaprochilodus kneri*		Yes	No	Yes
*Semaprochilodus laticeps*	*Jakato*	Yes	No	Yes
*Astyanax abramis*	*–*	Yes	No	Yes
*Astyanax bimaculatus*	*–*	Yes	No	Yes
*Leporinus friderici*		Yes	No	Yes
*Leporinus fasciatus*	*Waracu*	Yes	No	Yes
*Leporinus Moralesi*		Yes	No	Yes
*Schizodon fasciatus*	*Capirrito*	Yes	No	Yes
*Laemolyta orinocensis*	*Doponito*	Yes	No	Yes
*Anostomus anostomus*	–*Doponito*	Yes	No	Yes
*Pseudanos gracilis*	*–*	Yes	No	Yes
*Crenuchus spilurus*		Yes	No	Yes
*Hemiodopsis microlepis*	*Yonato*	Yes	No	Yes
*Hemiodopsis semifasciatus*	*Yonato–*	Yes	No	Yes
*Hemiodus gracilis*	*–*	Yes	No	Yes
*Bryconops alburnoides*	*–*	Yes	No	Yes
*Bryconops caudomaculatus*		Yes	No	Yes
*Chalceus macrolepidotus*	*Dopuento*	Yes	No	Yes
*Apistogramma borellii*	*Kaleito*	Yes	No	Yes
*Metynnis luna*	*Felefelwato*	Yes	No	Yes
*Metynnis argenteus*	*Janeri*	Yes	No	Yes
*Hemigrammus gracilis*	*Yaroto*	Yes	No	Yes
*Hemigrammus micropterus*	*–*	Yes	No	Yes
*Hemigrammus newboldi*		Yes	No	Yes
*Hemigrammus rhodostomus*	*Walabeto*	Yes	No	Yes
*Hemigrammus unilineatus*	*–*	Yes	No	Yes
*Mylossoma aureum*	*–*	Yes	No	Yes
*Mylossoma duriventre*	*–*	Yes	No	Yes
*Markiana geayi*	*–*	Yes	No	Yes
*Microschemobrycon casiquiare*	*–*	Yes	No	Yes
*Moenkhausia chrysargyrea*	*–*	Yes	No	Yes
*Moenkhausia copei*	*–*	Yes	No	Yes
*Moenkhausia cotinho*	*–*	Yes	No	Yes
*Moenkhausia grandisquamis*	*–*	Yes	No	Yes
*Moenkhausia megalops*	*–*	Yes	No	Yes
*Moenkhausia intermedia*	*–*	Yes	No	Yes
*Moenkhausia oligolepis*	*–*	Yes	No	Yes
*Triportheus angulatus*	*–*	Yes	No	Yes
*Triportheus orinocensis*		Yes	No	Yes
*Apistogramma ortmanni*	*Toseto*	Yes	No	Yes
*Brycon falcatus*	*Buejato*	Yes	No	Yes
*Brycon whitei*	*Wewerto*	Yes	No	Yes
*Brycon siebenthalae*	*Kuejeto*	Yes	No	Yes
*Brycon melanopterus*	*Yomatito*	Yes	No	Yes
*Brycon pesu*		Yes	No	Yes
*Roeboides affinis*	*jawasirto*	Yes	No	Yes
*Roeboides microlepis*	*Camalito*	Yes	No	Yes
*Heterocharax macrolepis*	*C*	Yes	No	Yes
*Tetragonopterus argenteus*	*Camalito*	Yes	No	Yes
*Tetragonopterus chalceus*	*Camalito*	Yes	No	Yes
*Markiana nigripinnis*	*Camalito*	Yes	No	Yes
*Brachyplatystoma rousseauxii*		Yes	No	Yes
*Calophysus macropterus*	*Tsawijana*	Yes	No	Yes
*Leiarius marmoratus*	*Tsawijana*	Yes	No	Yes
*Pimelodus blochii*	*Tsaliuto*	Yes	No	Yes
*Pimelodus clarias*	*Tsaliuto*	Yes	No	Yes
*Pimelodus pictus*	*Tsaliuto*	Yes	No	Yes
*Pseudoplatystoma tigrinum*	*Bunuju*	Yes	No	Yes
*Zungaro zungaro*	*Paletono/sipari*	Yes	No	Yes
*Aequidens chimantanus*	*Upeto*, *kaleito*	Yes	No	Yes
*Aequidens diadema*		Yes	No	Yes
*Aequidens tetramerus*	*Upeto*	Yes	No	Yes
*Astronotus ocellatus*	*Cabayuto*	No	No	Yes
*Salminus hilarii*	*Wewerto*	No	No	Yes
*Mikrogeophagus ramirezi*	*Kaleito*	No	No	Yes
*Cichla temensis*	*Bofala*	No	No	Yes
*Cichlasoma psittacum*	*Toseto*	No	No	Yes
*Caquetaia kraussii*	*Kaleito*	No	No	Yes
*Geophagus abalios*	*Upeto*	No	No	Yes
*Colossoma macropomum*	*Katzama*	No	No	Yes
*Piaractus brachypomus*		No	No	Yes
*Pristobrycon striolatus*	*Sirribo*	No	No	Yes
*Synbranchus marmoratus*	*Watsupi*	No	No	No
*Hypostomus punctatus*	*Tzama*	No	No	Yes
*Sorubim lima*	*Karresobo*	No	No	Yes
*Batrochoglanis villosus*	*Multo*	No	No	Yes
*Bryconamericus cismontanus*	*Yepe*	No	No	Yes
*Serrasalmus rhombeus*	*Catzama/tatama*	No	No	No
*Serrasalmus notatus*	*Sirribo*	No	No	No
*Acestrorhynchus falcirostris*	*Kuejeto*	No	No	Yes
*Acestrorhynchus falcatus*	*Awirri*	No	No	Yes
*Acestrorhynchus microlepis*	*Agujombo*	No	No	Yes
*Acestrorhynchus nasutus*	*Patirribo*	No	No	Yes
*Rhaphiodon gibbus*	*Patirribo*	No	No	Yes
*Hydrolycus scomberoides*	*Wemai*	No	No	Yes
*Hydrolycus tatauaia*		No	No	Yes
*Hoplerythrinus unitaeniatus*	*Enowü*	No	No	Yes
*Hoplias malabaricus*	*Tsumera*	No	No	Yes
*Boulengerella cuvieri*	*–*	No	No	Yes
*Boulengerella lateristriga*		No	No	Yes
*Boulengerella lucius*	*Tsutsubo*	No	No	Yes
*Boulengerella maculata*	*Tutsipabo*	No	No	Yes
*Ochmacanthus alternus*	*Wena*	No	No	Yes
*Acestridium colombiensis*	*–*	No	No	Yes
*Acestridium martini*		No	No	Yes
*Rineloricaria magdalenae*	*Bosikito*	No	No	Yes
*Rineloricaria formosa*	*Bosikito*	No	No	Yes
*Farlowella vittata*	*Tzutzubo*	No	No	Yes
*Loricaria cataphracta*	*Bosikito*	No	No	Yes
*Panaque cochliodon*	*Werria*	No	No	Yes
*Hypostomus ammophilus*	*Tzama*	No	No	Yes
*Hypostomus plecostomus*	*Tzama*	No	No	Yes
*Hypostomus watwata*	*Tzama*	No	No	Yes
*Pterygoplichthys gibbiceps*	*Tzama*	No	No	Yes
*Guianacara geayi*	*Tzama*	No	No	Yes
*Pterygoplichthys punctatus*	*Tzama*	No	No	Yes
*Paulicea lutkeni*	*Multo*	No	No	Yes
*Batrochoglanis raninus*	*Torito*	No	No	Yes
*Rhamdia wagneri*	*Rakiraki*	No	No	Yes
*Agamyxis albomaculatus*	*Yayakato*	No	No	Yes
*Platydoras costatus*	*Rakiraki*	No	No	Yes
*Hoplosternum littorale*	*Kokoto*	No	No	Yes
*Hydrolycus armatus*	*Tátama*	No	No	Yes
*Megalodoras irwini*	*Tátama*	No	No	Yes
*Orinocodoras eigenmanni*	*Yayakato*	No	No	Yes
*Ageneiosus inermis*		No	No	Yes
*Crenicichla geayi*	*Bopi*	No	No	Yes
*Crenicichla Johanna*	*–*	No	No	Yes
*Crenicichla lugubris*		No	No	Yes
*Crenicichla saxatilis*	*Bajiwi*	No	No	Yes
*Mesonauta egregius*	*Toseto*	No	No	Yes
*Curimata spilura*	*Kerpainto*	No	No	Yes
*Curimatopsis evelynae*	*Upato*	No	No	Yes
*Cichlasoma severum*	*Mamarto*	No	No	Yes
*Brachyplatystoma filamentosum*	*Pelsi*	No	No	Yes
*Brachyplatystoma juruense*	*Kajuyali topa*	No	No	Yes
*Brachyplatystoma vaillantii*	*–*	No	No	Yes
*Pseudoplatystoma fasciatum*	*Pelsi*	No	No	Yes
*Phractocephalus hemiliopterus*	*Dome*	No	No	Yes
*Platynematicthys notatus*	*–*	No	No	Yes
*Sorubimichthys planiceps*		No	No	Yes
*Electrophorus electricus*	*Kubebe*	No	No	No
*Cichla nigrolineatus*	*Bofala*	No	No	Yes
*Oxydoras niger*	*Jorojoro*	No	No	Yes
*Potamotrygon hystrix*	*Tulupo pone*	No	No	Yes

### Menarche

In Sikuani culture, the first and most significant stage of female life cycle is the menarche, as this moment of a woman’s life is a learning process in which the child acquires capabilities to play the roles of an adult. This knowledge is transmitted by the girl’s mother and grandmother and involves the preparation of traditional foods such as cassava bread and *yucuta* (drink made from a type of mandioca), the management of home and family issues, among other skills.

The menarche renders the girl more susceptible to become sickened by the influence of *ainawi*, for this reason fish consumption is completely avoided by the girl until a ritual called the prayer of the fish is performed, usually 15 to 90 days after the onset of the first menstruation. Although the prayer of the fish is how this ceremony is commonly translated, it narrates the Sikuani creation myth not only of fish, but also of amphibians, reptiles, birds, and mammals that belong to *ainawi* spirits. In Sikuani language, the prayer of the fish is called *Dujai matabajiwa* that actually signifies the prayer of the *ainawi*. About the prayer, a leader of the community said “what the shaman communicate to the *ainawi* is that there is one more person who is going to feed on fish and ask for protection”. The prayer of the fish consists of a chant that mentions 250 animals of aquatic habitat, according to the interviewees, in order to make an agreement with the *ainawi* to prevent the girl from being captured by them and to prevent disease. In the prayer of the fish, the girl’s family invites relatives and the people of the region to celebrate with food, chants, and dances. Before the ritual begins, men of the girl’s family fish as many species as possible. Women prepare and cook the fish to be prayed by the Sikuani shaman. During the prayer, all the fish of Sikuani regions are mentioned, from the smallest to the largest. After the prayer is done, the girl can eat fish, even when her next menstrual periods come. According to Sikuani tradition, when girls do not have the ritual or disrespect the dietary restrictions of menarche become more vulnerable to ailments caused by *ainawi*, a situation reminded by the myth of *Bakatsolowa*, a Sikuani story about a girl during her menarche who became a mermaid because of disobeying the restrictions related to fish consumption.

### Menstruation

During menstruation, the consumption of piranha fish of the genus *Serrasalmus* is restricted, since it is associated with abundant menstrual bleeding due to the presence of sharp teeth and reddish colour on its pectoral and abdominal areas. In general, the interviewees affirmed that during menstruation, there are no other restrictions for the consumption of fish, since the prayer constitutes lifelong protection that allows women to eat all the fish species consumed by others in the community.

### Gestation

It was found that among 139 fish species recognised as part of the Sikuani traditional diet, 66 were reported to be eaten during pregnancy while 73 are restricted. According to interviewees, fish with sharp teeth should not be eaten during pregnancy because they can “cut the umbilical cord with their teeth” and are associated with a higher occurrence of abortions. Piranha fish called in Sikuani *sirribo* and *catzama* (*Serrasalmus rhombeus*, *Serrasalmus notatus*, *Pristobrycon striolatus*) as well as *jorojoro* (*Megalodoras irwini, Oxidoras niger*), *rakiraki* (*Platydoras costatus*), and *yayakato* (*Orinocodoras eigenmanni*), which not only have sharp teeth but also have large fins, are considered to “detain the baby during labour”. Small fish characterised by pointed tail or mouth are restricted during pregnancy since they are regarded as abortive, these include the dog toothed fish called *kuejeto* in Sikuani (*Acestrorhynchus falcirostris*) and the needlefish or *tzuzubu* in Sikuani (*Farlowella vittata*).

The intake of *Tzama* fish that has plaques on the skin is associated with an excessive sensation of heat at night and insomnia of the pregnant woman, it may also cause the baby to suffer from skin problems. The *turuno* or *multo* in Sikuani (*Paulicea lutkeni*) is a large omnivorous fish that can “take away the soul and cause death” of babies when eaten by pregnant women. The consumption of the electric eel or *kubebe* (*Electrophorus electricus*) is restricted during pregnancy because of its cylindrical and snake-like shape; however, its burned bone is used to facilitate labour because the fish skin is very smooth, and this feature is associated with a rapid delivery.

According to the interviewees, the consumption of certain fish can have effects on the physical characteristics and personality of newborns. For example, *bajiwi* are small fish with a spot on its tail peduncle that resembles an eye, and they should not be eaten during pregnancy because their consumption is associated with babies born with red eyes who become shy in the future. Similarly, if the pregnant woman eats a medium-sized fish with pointed teeth called *dormilon*, *guabina*, or *tsumera* in Sikuani (*Hoplerythrinus unitaeniatus*), it is associated with infants that eat rubbish when start to crawl.

### Principal component analysis

**Fig. 2:**
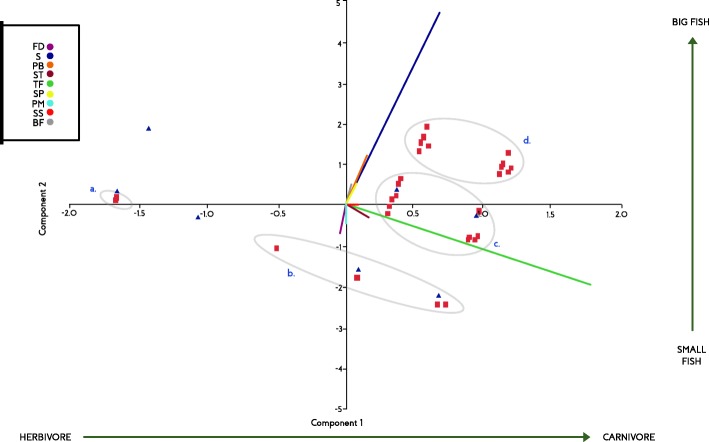
PCA of morphological features of fish and selection or restriction on diet during pregnancy. TF, type of food; S, size; ST, sharp teeth; BF, big fins; PB, presence of barbels; PM, peaked mouth or acuminate caudal peduncle; FD, fish that disguise or presence of spot at the caudal peduncle; PP, skin in plates; SS, snake shape. Blue triangles represent edible fish and red squares restricted fish during pregnancy. Groups A, B, C, and D delimited in ovals represent most of the species restricted during pregnancy. Green axis represents PC1 and blue axis PC2

According to PCA results, the principal component 1 (PC1) explained 74.96% of the data variation. The most important variables of PC1 were the type of feeding and size of the fish (eigenvalues 0.9 and 0.35 respectively) and therefore are the most determining characteristics for dietary avoidance or allowance of fish during pregnancy. In PC2, the most important variables were the size of the fish and the presence of barbels (eigenvalues 0.88 and 0.22 respectively). In Fig. 2, PC1 is represented on the *X* axis and in PC2 on the *Y* axis. The fish considered edible during pregnancy are represented with blue triangles, while the restricted ones with red squares. The fish avoided during pregnancy correspond mainly to big carnivorous or omnivorous fish that can have barbells on their jaw, these are included in groups C and D. However, group C also includes two blue triangles that represent medium-sized but carnivorous or omnivorous fish with barbells that are eaten during gestation. The majority of small fish are eaten during pregnancy, with the exception of those that have a spot on their caudal peduncle or a pointed mouth or tail (groups A and B).

### Postpartum

The period between birth and the next 40 days postpartum is very important for Sikuani people since the baby’s health and vitality depend on the care received during this period of time. At this stage, the parents use to limit their duties; the father must neither hunt nor work and the woman must designate her housework to other women in the community such as her mother or sisters, since otherwise the navel of the newborn can bleed. During the first days of postpartum, the woman and her husband avoid fish consumption until the prayer of the fish takes place. In this occasion, the ritual is not performed as a collective celebration, it is an intimate setting. Once the child begins, the complementary diet, the prayer of the fish is made to introduce the new member of the community to the *ainawi*, in order to prevent the disease.

## Discussion

### Fish size, trophic level, and toxicity

According to Sikuani traditional knowledge, there are relations between fish consumption and woman health along the reproductive cycle. Sikuani women avoid eating certain fish during menarche, menstruation, pregnancy, and postpartum as its consumption is related with traditional ailments. According to the results of this study, the fish size and its feeding habits are the most important characteristics associated with the avoidance of its consumption by pregnant Sikuani women. Regarding the feeding habits, carnivorous fish are not eaten during pregnancy, compared to herbivores and insectivorous species, which are eaten unless they have other specific banning characteristics. Pregnant Sikuani women also abstain from eating sharped-teeth fish as its consumption is associated with abortions; this morphological feature is commonly found in carnivorous fish. Previous studies have reported that inhabitants of Brazilian Amazon and Atlantic coast during the menstruation and the puerperium, as well as when recovering from diseases as injuries or wounds, avoid the consumption of carnivorous fish, as well as fish of aggressive behavior, hard flesh, high-fat content, or without scales, preferring herbivorous, insectivorous, and detritivorous species [29].

Large and carnivorous fish are at higher trophic levels than smaller and non-carnivorous ones. It has been evidenced that animals at upper levels of the food chain, such as carnivorous fish, tend to bioaccumulate toxins like heavy metals or plaguicides from eating plants and animals at lower trophic levels. Pesticides like DDT (dichloride diphenyl trichloroethane) have been detected in fish flesh in levels 10 times higher when it is ingested through the diet rather than through water [30]. Carnivorous fish also bioaccumulate greater amounts of heavy metals than omnivorous, detritivores, insectivorous, and herbivorous species. Previous studies about mercury, methylmercury, lead, cadmium, and metalloid arsenic concentrations on fish from Colombian rivers have evidenced higher levels of these heavy metals in the flesh of carnivorous fish when compared to non-carnivorous ones [31–33]. The amount of heavy metals on Colombian fish exceed the maximum levels recommended by the World Health Organisation and the consumption of fish from mining regions have been directly associated with high concentrations of heavy metals detected in humans [34]. The regulation of carnivorous fish consumption by Sikuani women during important life stages may have beneficial effects on health, decreasing the intake of toxins accumulated on these species. The avoidance of carnivorous fish by inhabitants of the Brazilian Amazonas and Atlantic Coast has been considered a strategy to adapt to changing environmental conditions and to preserve health [29].

### Signatures of ainawi

During menarche, menstruation, pregnancy, and postpartum Sikuani women regulate the consumption of fish, because it is considered to cause illness if *ainawi*, as protector spirits of water and animals are not asked for permission previously. Other American indigenous groups like Mapuche, Quechua, and Aymara people also believe in the existence of spiritual beings who are considered protectors or owners of all-natural resources, entities that must be requested for permission when humans plan to visit their habitats or eat animals [35–38].

Most of the regulations of fish consumption during pregnancy among Sikuani women may be interpreted as associations of similarity conceived between the morphology, diet and other aspects of fish, and the effects they generate on women and foetus health. According to informants, these fish features are traditionally comprehended as an expression of the force of its *ainawi* and its potency to cause disease to humans. This reasoning may be analysed using the theories of sympathetic magic and the doctrine of signatures. Sympathetic magic underlies diverse traditions, rituals, and conceptions; it operates through the law of contagion in which contacted objects transfer some of their properties, eliciting consequences that may be temporary or permanent. The doctrine of signatures holds that similar objects may exert an action or effect related to their shapes or functions, as might be the case of certain foods that are avoided because they resemble undesirable characteristics or conditions [39]. The interviewees consider the *ainawi* of fish may persist after eating them and are able to provoke health problems to the pregnant woman and the foetus, evidencing concepts of contagion [39]. It is also stated by the informants that some characteristics of fish give information about the health conditions or ailments they can induce, features conceived as signatures of *ainawi* on fish. In a similar way, other indigenous communities believe animals can transfer their characteristics to people who eat them. As referred by Sikuani people from Wacoyo Reservation, the shakerfish (*Electrophorus electricus*) has been also used to facilitate deliveries in Spanish ethnomedicine [40]. Some communities from Ghana circumvent the intake of snakes during pregnancy, as it is associated through similarities with dry scaly skin in the newborn [41]. At the Peruvian Amazon, the speakers of Iquitos language consider the meat of fish and mammals with sharp teeth may be harmful to people recovering from disease, as it resembles the magical darts used by witches to attack their victims [42].

### Prayers to ainawi

Sikuani people consider the prayer of the fish a formal introduction of community members to the spirits of nature during important life stages; in order to prevent *ainawi*-associated ailments. The fish features associated with disease are reflected on the prayer of the fish, as the first fish to be referred at the beginning of the myth are the smallest ones and are almost always considered edible for Sikuani women, while large and carnivorous fish are mentioned at the end of the prayer and are more commonly forbidden during specific stages of life. Since this point of view, the order of appearance of fish on the myth can be related with the symbolic introduction of animals into Sikuani diet after menarche or birth, beginning with the less harmful ones and ending with animals whose consumption is closely related to *ainawi* ailments.

The prayer of the fish ceremony is related to the Sikuani story of *Bakatsolowa*, a girl who became a mermaid after eating fish without the ritual of menarche. This myth refers to girls’ vulnerability of being captured and abducted into the underworld or fish world by the *ainawi*. This narration acts as a reminder in Sikuani culture about the restrictions on fish consumption during specific moments of the life cycle or when recovering from disease, and also about the importance of the prayer of the fish as a traditional preventive measure.

Likewise, other communities from different regions of the world have similar food regulations and rituals associated with the first menstruation, as is the case of a traditional community in Sri Lanka in which during menarche and while the menstrual bleeding lasts, the girl receives a diet without fish, oils, and spices to protect her from future diseases. Once the restriction ends, there is a celebration with relatives, friends, and neighbors where several dishes are served, including those previously avoided by the girl [43].

### Diet regulations and socio-ecological health and resilience

Sikuani people consider the avoidance of certain foods at specific stages of life and the associated rituals like the prayer of the fish, are traditional approaches to preserve human health, to maintain the balance of ecosystems, to prevent natural resources extinction, and to reinforce social cohesion in their communities. The majority of regulations upon fish consumption during menarche, menstruation, pregnancy, and postpartum among Sikuani women from Wacoyo Reservation are directed toward prevention of woman disease, problems during childbirth and diseases, malformations in the newborn or other *ainawi*-associated ailments. Health reasons for food avoidance are also reported in other communities, the Kalenjin women from Kenya evade the consumption of certain foods to prevent neonatal death, skin lesions, abortions, and premature birth [44]. Women from Myanmar avoid during the postpartum to eat certain kinds of fish considered not suitable for mothers’ and babies’ health [45].

Some authors state that restrictions for the utilisation of natural resources among traditional societies can be understood as ecological adaptations to maintain the resilience of ecosystems and social groups [46]. The relationship that Sikuani communities have established with nearby rivers fish, transcends the physical and involves an interaction between nature and culture, between supernatural and human [47]. The Sikuani of the Wacoyo Reservation have managed to conserve their traditions despite the significant reduction of their territory due to appropriation of their ancestral lands by settlers [7]. Nowadays, these dietary customs are changing and might disappear, as most of health and nutrition conceptions or practices of Sikuani people are frequently ignored or rejected at hospitals or other institutions; an issue threatens the use of indigenous foods and provokes a progressive decline of traditional food systems [4]. The inclusion of traditional Sikuani knowledge in the formulation and implementation of policies involving their communities may constitute an important contribution to develop intercultural strategies to solve health issues and for the sustainable management of natural resources.

## Conclusions

The traditional diet of Sikuani women includes numerous fish species and an important proportion of them are avoided during menarche, menstruation, gestation, and postpartum. It was found that during menarche and postpartum, fish consumption is avoided by Sikuani women only before the ritual known as the prayer of the fish is performed. The menstruation does not imply significant regulations for fish intake, while during pregnancy there are multiple and specific avoidances for consumption of fish. According to our results, there are some features of fish associated with their regulation on the diet of pregnant Sikuani women, who evade to eat mainly carnivorous, large, or sharp teeth fish, as well as fish with plaques, pointed mouth, or tail or with a spot on their caudal peduncle. The consumption of these kinds of fish is avoided during pregnancy because it is related to the appearance of disease caused by *ainawi*, protector spirits of aquatic animals. The aim of the traditional Sikuani ceremony called prayer of the fish is to intercede with the *ainawi* to ask for protection for the members of the community that feed on fish and other aquatic animals. The regulations among Sikuani people for the consumption of carnivorous fish may be related to their higher trophic level and with the fact these species accumulate a greater amount of toxins like plaguicides and heavy metals. The main reasons underlying the avoidance of fish consumption by Sikuani women are the prevention of human disease as well as the strengthening of communities and ecosystems resilience. Further studies to evaluate the impacts on nutrition of traditional foods and the effects of regulations for their consumption among Sikuani people are recommended.

## Data Availability

Additional data on the interviews is available from the first author upon request.

## Abbreviations

PCA: Principal component analysis
